# Development of an automated image analysis protocol for quantification of intracellular forms of *Leishmania* spp.

**DOI:** 10.1371/journal.pone.0201747

**Published:** 2018-08-02

**Authors:** Ana G. Gomes-Alves, André F. Maia, Tânia Cruz, Helena Castro, Ana M. Tomás

**Affiliations:** 1 i3S – Instituto de Investigação e Inovação em Saúde, Universidade do Porto, Porto, Portugal; 2 IBMC – Instituto de Biologia Molecular e Celular, Universidade do Porto, Porto, Portugal; 3 CEB – Centro de Engenharia Biológica, Universidade do Minho, Braga, Portugal; 4 ICBAS – Instituto de Ciências Biomédicas Abel Salazar, Universidade do Porto, Porto, Portugal; Academic Medical Centre, NETHERLANDS

## Abstract

*Leishmania* parasites cause a set of neglected tropical diseases with considerable public health impact, the leishmaniases, which are often fatal if left untreated. Since current treatments for the leishmaniases exhibit high toxicity, low efficacy and prohibitive prices, many laboratories throughout the world are engaged in research for the discovery of novel chemotherapeutics. This entails the necessity of screening large numbers of compounds against the clinically relevant form of the parasite, the obligatory intracellular amastigote, a procedure that in many laboratories is still carried out by manual inspection. To overcome this well-known bottleneck in *Leishmania* drug development, several studies have recently attempted to automate this process. Here we implemented an image-based high content triage assay for *Leishmania* which has the added advantages of using primary macrophages instead of macrophage cell lines and of enabling identification of active compounds against parasite species developing both in small individual phagolysosomes (such as *L*. *infantum*) and in large communal vacuoles (such as *L*. *amazonensis*). The automated image analysis protocol is made available for IN Cell Analyzer systems, and, importantly, also for the open-source CellProfiler software, in this way extending its implementation to any laboratory involved in drug development as well as in other aspects of *Leishmania* research requiring analysis of *in vitro* infected macrophages.

## Introduction

Trypanosomatid protozoan parasites of the genus *Leishmania* are the causative agents of some of the most significant neglected tropical diseases, the leishmaniases. There are over 20 species of *Leishmania* which, together with the particular genetics and immune characteristics of infected individuals, can cause a variety of clinical manifestations, including cutaneous and mucocutaneous forms and a fatal visceral condition [1]. The leishmaniases are present in 98 tropical and subtropical countries, where approximately 1 billion people are at risk of infection [1,2]. One alarming reality in such a scenario is the lack of adequate anti-*Leishmania* drugs, as current therapeutics exhibit important drawbacks like high toxicity, low efficacy, difficult administration and prohibitive prices [3]. Furthermore, the long treatment regimens of most drugs together with the side effects they cause bring about low medication compliance which, in turn, promotes disease relapse and emergence of drug resistant parasite strains [4]. Even though combination therapy, as encouraged by World Health Organization, circumvents some of those problems, the development of novel and more adequate anti-*Leishmania* treatments remains urgent [5]. Whatever the exact strategy followed in drug discovery [6], this process is much facilitated by the capacity to rapidly screen *in vitro* many compound library series against *Leishmania* parasites through automated platforms. To reproduce the physiological conditions encountered *in vivo*, such high-throughput screening (HTS) assays should use the clinically relevant parasite stages and host cells.

Infection by *Leishmania* takes place when female sandflies inoculate promastigotes into a mammalian host during a blood meal. Promastigotes are phagocytosed by mononuclear phagocytic cells, mainly macrophages, and differentiate into amastigotes; after replication in phagolysosomes, the latter infect new cells [7,8]. The intracellular amastigote is therefore the disease-causing stage of *Leishmania* and, as such, the target that high throughput campaigns must aim at. Although *Leishmania* can invade different phagocytic cells, only macrophages support replication. Previously developed screening assays often used the human monocytic leukemia THP-1 and other cell lines as models as these are easy to handle and have high proliferative rates providing an unlimited source of material [9]. Nevertheless, usage of cell lines for screening purposes is not without concerns. Apart from cross-contamination with other cell lines and with mycoplasma [10], the genotypic and phenotypic changes that can arise due to continuous passages *in vitro* may lead to important differences relative to the primary cells that they represent [9], affecting overall results. An alternative option is the use of primary macrophages that, despite having a limited lifespan, maintain most *in vivo* physiological characteristics and functions.

The purpose of this study was twofold. First, to implement a high content analysis (HCA) protocol that used primary macrophages as host cells for *Leishmania* amastigotes, to identify new therapeutic drug candidates for all types of leishmaniases and facilitate other studies requiring parasite enumeration in *in vitro* macrophage infections. Second, to make available high throughput quantification of infection assays using microscopy images obtained by any kind of fluorescence microscope and without the need of expensive image analysis software. For this, we provide the image-analysis pipeline developed during this work on the free open-source cell image analysis software, CellProfiler [11].

## Materials and methods

### Parasites

Promastigotes of *L*. *infantum* (MHOM/MA/67/ITMAP-263) and *L*. *amazonensis* (MHOM/BR/LTB0016) were cultured at 25°C in RPMI 1640 Glutamax supplemented with 10% (v/v) heat inactivated fetal bovine serum (iFBS), 50 U ml^-1^ penicillin, 50 μg ml^-1^ streptomycin (from Gibco) and 20 mM HEPES pH 7.4 (Sigma) and in Schneider’s Insect medium (Sigma) supplemented with 10% (v/v) iFBS, 100 U ml^-1^ penicillin, 100 μg ml^-1^ streptomycin, 5mM HEPES pH 7.4 and 5μg ml^-1^ phenol-red (Sigma) media, respectively. Promastigotes of both species were passaged through mice in order to ensure infectivity. Axenic amastigotes of *L*. *infantum* were differentiated from parasites recently recovered from the spleen of infected mice, as described before [12], and maintained at 37°C, 5% CO_2_, in MAA medium supplemented with 20% (v/v) iFBS, 2 mM Glutamax (Gibco), and 0.023 mM hemin (Sigma).

### Macrophages

Bone marrow-derived macrophages (BMDM) were generated by adapting the protocol described by Gomes et al. [13]. Briefly, bone-marrow cells, collected by flushing femurs and tibia of BALB/c mice with Hank’s Balanced Salt Solution (HBSS), were centrifuged and suspended in Dulbecco’s Modified Eagle’s Medium (DMEM) supplemented with 1% (v/v) Minimum Essential Medium Non-Essential (MEM) amino acids solution (from Gibco), 10% (v/v) iFBS, 50 U ml^-1^ penicillin, 50 μg ml^-1^ streptomycin (cDMEM) and 10% (v/v) L929 cell conditioned medium (LCCM) as a source of macrophage colony-stimulating factor. Bone-marrow cells were placed in Petri dishes and incubated at 37°C with 5% CO_2_. Twenty four hours later, non-adherent cells were collected, counted, plated in 96-well plates (2.5 to 3x10^4^ cells per well) and incubated at 37°C with 5% CO_2_ for 10 days, with cDMEM+10%LCCM renewal on the 4th and 7th days.

### Infections and drug assessment

Bone marrow-derived macrophages, in 96 well plates, were infected with *L*. *infantum* axenic amastigotes (3-days culture) or *L*. *amazonensis* stationary promastigotes (5-days culture) at a multiplicity of infection (MOI) of 10. After 3 hours of contact with macrophages, non-phagocytosed parasites were removed by washing twice with cDMEM. Internalized parasites were allowed to differentiate into amastigotes for 24 hours prior to being challenged with serially diluted Amphotericin B (Sigma) or vehicle (DMSO). Twenty four hours after addition of the compounds, cultures were fixed with paraformaldehyde and processed for HCA.

### Identification of parasites and macrophages in infection assays

Intramacrophagic *Leishmania* amastigotes were identified upon staining of their DNA with 4′,6-diamidino-2-phenylindole (DAPI; Sigma). Macrophages were labelled with high-content screening (HCS) CellMask^™^ Deep Red stain (Invitrogen) which labels both nucleus and cytoplasm, hence providing a means to delineate the cell boundary. Both, DAPI and HCS CellMask^™^ were added together to cells previously fixed with 4% paraformaldehyde (w/v) and permeabilized with 0.1% (v/v) Triton X-100 (Sigma), at room temperature for 30 minutes. For visualization of intracellular parasites with an indirect fluorescent antibody test (IFAT), fixed and permeabilized cultures were blocked with 1% (w/v) BSA and then incubated with a mixture of anti-cTXNPx1 [14] and anti-mTPx [14] as primary antibodies, and an Alexa Fluor 568 anti-rabbit IgG (Molecular Probes) as secondary antibody, all used at 1:2000 dilution at room temperature for 2hrs.

### Image acquisition

Images were acquired in an IN Cell Analyzer 2000 microscope (GE Healthcare) with a Nikon 20x/0.45 NA Plan Fluor objective (binning 1X1), using a large chip CCD Camera (CoolSNAP K4) with a pixel array of 2048x2048 (7.40 μm^2^ pixel). Image field of view (FOV) x-y for this objective is 0.76x0.76 mm. With these settings and an approximate 70–80% cell confluence, 1500–2000 macrophages per well were imaged (per 4 FOV), a number we consider reasonable for the quantifications as it is 15 to 20-fold higher than that used with manual counting [15–17]. The excitation and emission filters used to detect DAPI, Alexa Fluor 568 and HCS CellMask^™^ were DAPI, Texas Red, and Cy5, respectively.

### Image analysis using the IN Cell Investigator Developer Toolbox

**Fig 1:**
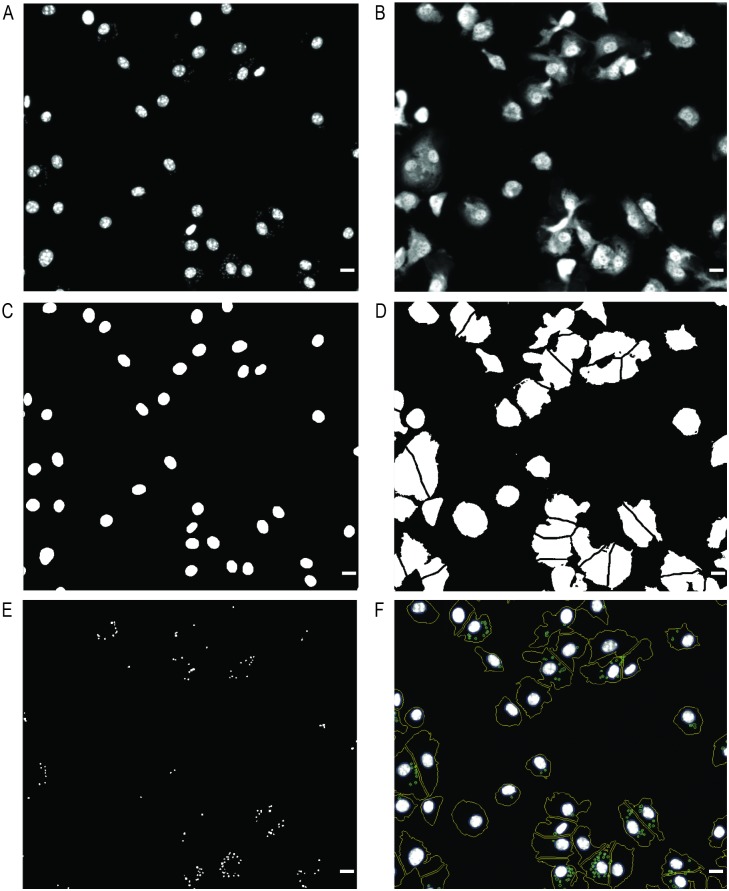
Detection of intramacrophagic parasites using IN Cell Investigator Developer Toolbox. Raw image from (A) DAPI and (B) HCS CellMask^™^ Deep Red channels acquired with IN Cell Analyzer 2000 microscope. (C) Nuclei of macrophages identified by a pre-defined nuclear segmentation for objects stained with DAPI larger than 50μm^2^. (D) Cytoplasm of macrophages recognized by HCS CellMask^™^ Deep Red staining. (E) Parasites are objects stained with DAPI localized inside cytoplasm and with sizes between 0.25–1μm. (F) Final processed image showing macrophage nuclei (blue line), cell boundaries (yellow line) and parasites (green line). Scale bar, 10μm.

Original images were analyzed with the IN Cell Investigator Developer Toolbox v. 1.9.2 (GE Healthcare) (Fig 1A and 1B). This image analysis workflow allows the identification of the host cell nuclei (a DAPI stained structure with a size greater than 50 μm^2^) and cytoplasm, as well as of the DNA content of parasites. For macrophage nuclei identification we used the DAPI channel (Fig 1A) and applied a pre-defined nuclear segmentation algorithm. This operation uses a variation of the ‘*top-hat’* approach to segmentation followed by a binary opening procedure to smooth the contours of the segmented nuclei and by a hole filling procedure. We then applied a slight expansion (dilation) of the nuclear mask in order to avoid incorrect detection of parasites (Fig 1C). The cell cytoplasm was segmented from the HCS CellMask^™^ raw image (Fig 1B) using an intensity segmentation operation. This operation employs simple multilevel global thresholding to classify a pixel as either belonging, or not, to a target set. In order to separate adjacent touching cells, we executed a ‘clump breaking’ procedure consisting of approximate cellular boundaries using the Voronoi tessellation generated from a ‘seed’ point, in this case a segmented nuclear image (Fig 1D). Parasites were also segmented from the DAPI raw image using a pre-defined granular segmentation algorithm. This operation uses a multi-scale *‘top-hat’* approach based on a minimum (0.25μm) and a maximum granule size (1.00μm), hence being easily distinguishable from macrophage nuclei. Only parasites located inside the cells were counted. Due to heterochromatin heterogeneity, macrophage nuclei often present a granular aspect and these granules can occasionally be confused with parasites. To avoid such “false positives”, the nuclear mask (Fig 1C) was subtracted from the parasite segmentation mask (Fig 1E). The final processed image is shown in Fig 1F, where macrophage nuclei, macrophage boundary and parasites are outlined in blue, yellow and green, respectively. Every segmentation algorithm used here can be fine-tuned through sensitivity adjustment to optimize the analysis of the images. Upon segmentation of all objects of interest, quantitative data were extracted. Accordingly, we obtained the total number of macrophages and parasites, the number of infected macrophages and the number of parasites per infected macrophage.

### Image analysis using CellProfiler

**Fig 2:**
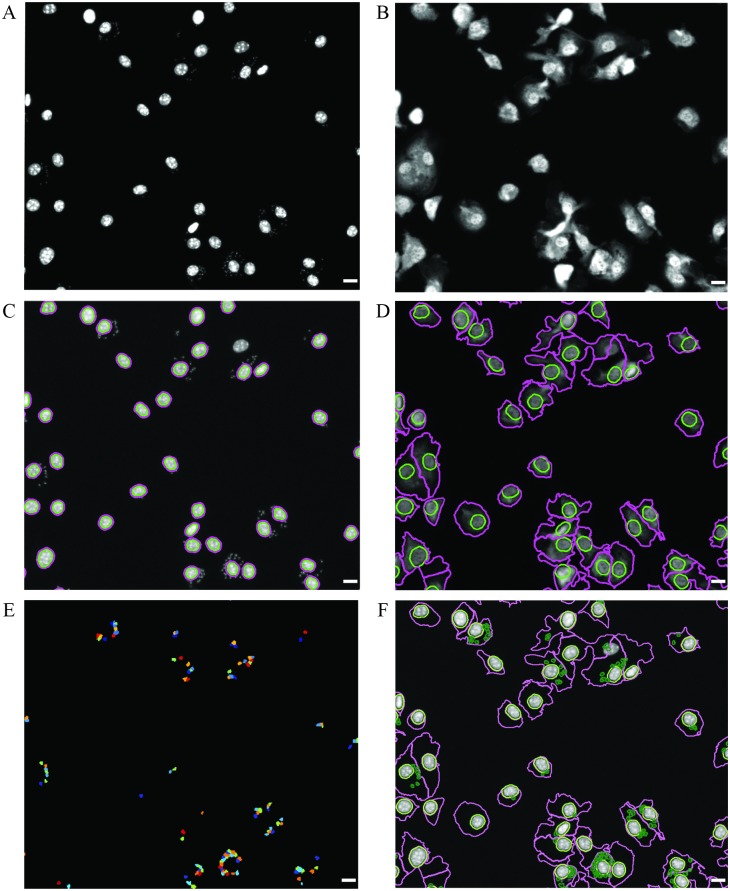
Detection of intramacrophagic parasites using Cell Profiler. Raw image from (A) DAPI and (B) HCS CellMask^™^ Deep Red imaging channels acquired with IN Cell Analyzer 2000 microscope. (C) BMDM nuclei identified as DAPI-positive objects with a size range of 18–50 pixels (green line); also depicted is the nuclear expansion applied to avoid false positive detection of parasites in the vicinity of the nuclei (pink line). (D) Macrophages detected by a propagation algorithm of the HCS CellMask^™^ Deep Red staining which starts in the nucleus (green line) and ends in the BMDM cell boundaries (pink line). (E) Parasites identified as DAPI-staining objects with 4–12 pixels located within the cytoplasm of BMDMs. (F) Final processed image showing BMDM nuclei expansion (light green line), cell boundary (pink line) and parasites (dark green line). Scale bar, 10μm.

The following specifications were used to analyze images with CellProfiler (2.2.0) (Fig 2A and 2B). For nuclei segmentation we applied a Global thresholding strategy followed by an Otsu thresholding method. Adjacent merged nuclei were separated into individual objects of interest by identification of a central single peak of brightness on each nucleus. Next, we did a slight expansion (dilation) of the nuclear mask in order to avoid false positive detection of parasites in the vicinity of the nuclei (Fig 2C). After this, the cell cytoplasm was segmented from the HCS CellMask^™^ raw image through a propagation algorithm of the mask starting from the previously identified nucleus until the edges of the staining using the same thresholding method as for nuclei segmentation (Fig 2D). Parasites were segmented using a similar methodology, based on a typical diameter of the parasites between 4–12 pixel units. Adjacent merged parasites were separated into individual objects through the identification of a central single peak of brightness on each organism (Fig 2E). Next, the number of amastigotes in the cytoplasm mask was calculated. The final processed image is shown in Fig 2F. The algorithm can be modified to optimize image analysis through fine-tuning of threshold correction factors. CellProfiler analysis provides the total number of macrophages and parasites, the number of infected macrophages and the number of parasites per infected macrophage.

### Ethical statement

BALB/c mice were obtained from the i3S animal facility. Animal procedures were approved by the Local Animal Ethics Committee of i3S, licensed by DGAV (Direção Geral de Alimentação e Veterinária, Govt. of Portugal—DGAV). Animals were handled in strict accordance with good animal practice as defined by national authorities (DGAV, directive 113/2013 from 7th August) and European legislation (directive 2010/63/EU, revising directive 86/609/EEC). The i3S animal house is certified by DGAV. Mice were euthanized by an overdose of isoflurane inhalation followed by cervical dislocation.

### Statistical analysis

Statistical analysis (One way ANOVA) to compare data extracted from CellProfiler and IN Cell Investigator Developer Toolbox with that obtained by manual counting was performed using GraphPad Prism Software 5.02. This same software was also used to calculate amphotericin B half maximal inhibitory concentrations (IC_50_).

## Results

### Parasite and infected cell labelling

**Fig 3:**
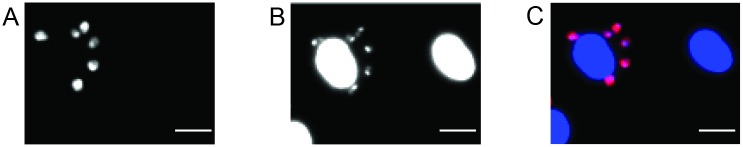
Intracellular *L*. *infantum* labeled with anti-parasite antibodies by IFAT and with DAPI. *Leishmania infantum*-infected BMDM stained with (A) anti-cTXNPx and anti-mTXNPx and with (B) DAPI (arrows identify intracellular parasites). (C) Merged image showing co-localization of DAPI-stained (blue) parasites with antibody labeling (red). Images were acquired with an IN Cell Analyzer 2000 microscope. Scale bar, 10μm.

**Fig 4:**
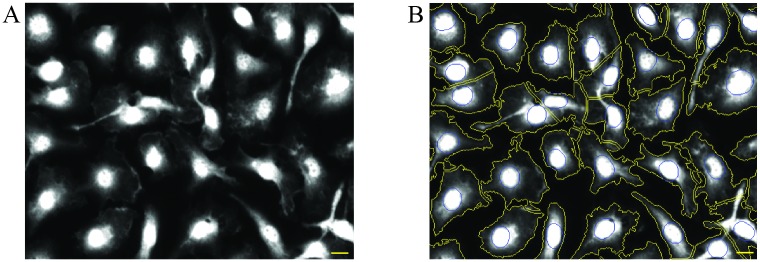
Heterogeneous morphology of BMDM. (A) HCS CellMask^™^ Deep Red raw image acquired with an IN Cell Analyzer 2000 microscope (B) Cytoplasm boundary defined by IN Cell Investigator Developer Toolbox (yellow line). Blue lines represents cell nucleus. Scale bar, 10μm.

In this work, we implemented HCS assays for quantification of intracellular *Leishmania* using primary murine macrophages as the parasite host cells. A crucial step in microscopy-based high content methodologies is the capacity to detect and discriminate between the different image elements accurately, in this case intracellular parasites and macrophages. Amastigote identification was achieved employing DAPI staining, as described previously [18–20]. The suitability of this stain was confirmed by labelling intracellular *L*. *infantum* with DAPI and with anti-parasite antibodies by IFAT (Fig 3). We used the anti-cTXNPx and anti-mTXNPx antibodies (Fig 3A), which recognize proteins located in the parasite cytoplasm and mitochondria, respectively. This approach showed that all structures detected with DAPI co-localized with the antibody-staining (Fig 3B and 3C). For macrophage detection both nuclei and the cytoplasm were taken into account. The former were identified with DAPI, the second by delimiting their contour. Some HCA studies have defined the macrophage boundary by computation based on the position of the nucleus [18,20]. This approach, that assumes that nuclei are consistently located in the center of the cell, can lead to inaccurate results especially when using primary macrophages such as BMDMs. Indeed, the morphology of these is not as homogeneous as that of macrophage cell lines, in many instances presenting protrusions that expand away from the nucleus (Fig 4). To overcome this issue, we have defined BMDM contour, as described by Manu De Rycker et al. [21], taking advantage of the HCS CellMask^™^, a cell delineation tool for HCS assays. As shown in Fig 4, this mask clearly labels the cell cytoplasm facilitating macrophage identification even in cultures with heterogeneous morphologies.

### IN Cell Investigator Developer Toolbox and CellProfiler image analysis

**Fig 5:**
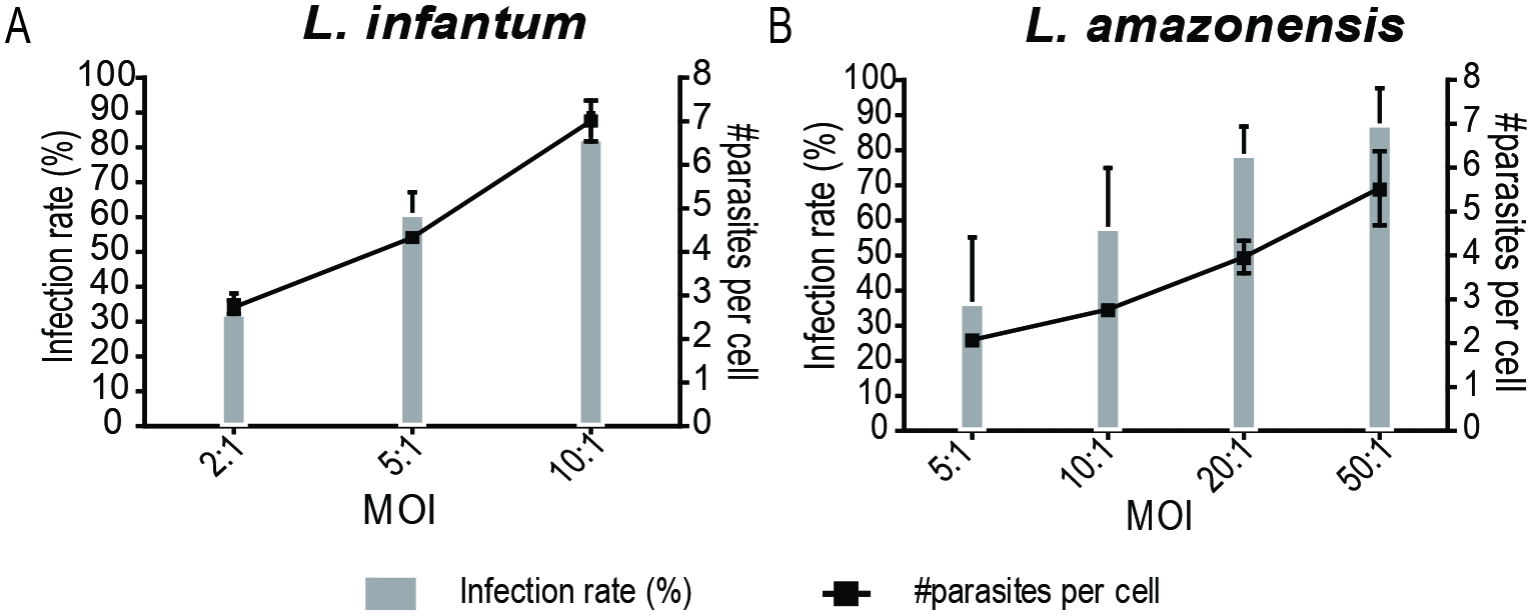
IN Cell Investigator Developer Toolbox infectivity parameters depends on the MOI. Infection rate and the number of intracellular amastigotes by (A) *L*. *infantum*, (B) *L*. *amazonensis* using different parasite:BMDM ratios (MOI), as detected by IN Cell Investigator Developer Toolbox. Values represent the mean and standard deviation of three independent experiments.

The first image protocol developed here used the IN Cell Investigator Developer Toolbox. The program identifies individual macrophages, by the association of a nucleus with a well-delimited cytoplasm, and the parasites within each of them. Two important algorithm features should be outlined. First, parasites that do not co-localize inside macrophage boundaries are not taken into account to calculate the percentage of infected macrophages and the number of intracellular parasites. Second, even though DAPI staining labels two different structures in each parasite, one corresponding to nuclear DNA and the other to the mitochondrial kinetoplast DNA (kDNA), the segmentation applied ensures that only the latter, which is brighter, is identified by the software algorithm as a parasite (Fig 1E). The ability of the software to correctly detect macrophages and intracellular *L*. *infantum* amastigotes is illustrated in Fig 1F and S1C Fig. Unlike *L*. *infantum*, that replicates in tight-fitting, individual phagolysosomes, some *Leishmania* species propagate within giant vacuoles hosting many parasites, a phenotypic characteristic that can reflect on the morphology of the macrophage. Using *L*. *amazonensis*, a representative of such species, we ensured that the type of phagolysosome parasitized by *Leishmania* does not interfere with the capacity of the program to correctly identify macrophages and parasites (S1C Fig), in this way validating the use of the algorithm to any *Leishmania* species. Fig 5, showing that the estimated infection rates augment as the multiplicity of infection (MOI) increases, also suggests that the system is adequate for intramacrophage amastigote quantification.

The IN Cell Investigator Developer Toolbox is a commercial, expensive, software that may not be available to all research groups. In order to enable usage of our framework by other labs working on *Leishmania*, we have adapted our image analysis workflow to an open-source software, CellProfiler [11], which permits analysis of images acquired by any kind of fluorescence microscope, including of high throughput. CellProfiler identifies macrophages and parasites similarly and as effectively as the IN Cell Investigator Developer Toolbox, as demonstrated for *L*. *infantum* (Fig 2F) and *L*. *amazonensis* (S1D Fig).

### Comparison of Cell Profiler with IN Cell Investigator Developer Toolbox

**Fig 6:**
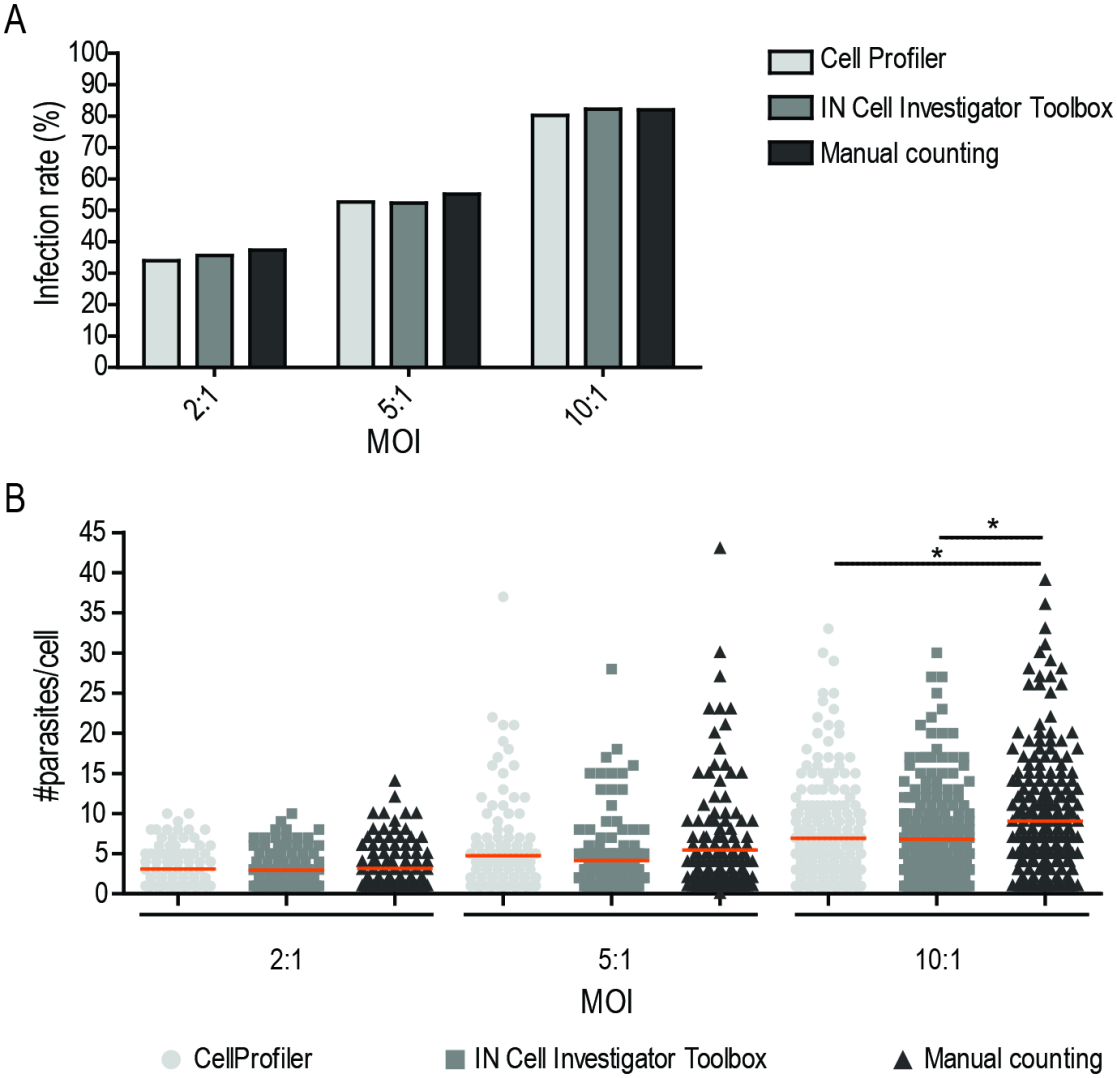
Comparison of IN Cell Investigator Developer Toolbox and CellProfiler image analysis protocols with manual counting. BMDMs were infected with *L*. *infantum* axenic amastigotes at different MOI. (A) Infection rate. (B) Number of parasites per infected cell obtained with each automated protocol was compared with manual counting. Each dot represents the number of parasites in a single host cell and horizontal lines show the mean. Around 300 cells counted in each MOI condition. Statistical analysis was performed using the One-Way ANOVA. P-values correspond to *p<0.05.

Validation of CellProfiler and IN Cell Investigator Developer Toolbox image analysis workflows was performed by comparing their data output with that obtained by manual counting (i.e., upon eye enumeration of macrophage and parasite numbers on the acquired images). For this purpose, the number of parasites detected in each case in approximately 900 *L*. *infantum*-infected macrophages (300 representative of each MOI) was registered. Using these data, we calculated the infection rate and the number of parasites per macrophage (Fig 6). We found that the former parameter was similar in all three methodologies (Fig 6A). As for the number of parasites per macrophage, this also compared well in the MOI of 2 and 5 but was found lower when using automated analysis in the MOI of 10 (Fig 6B). This occurs due to a greater number of highly infected macrophages in this condition making discrimination of single parasites less accurate.

**Fig 7:**
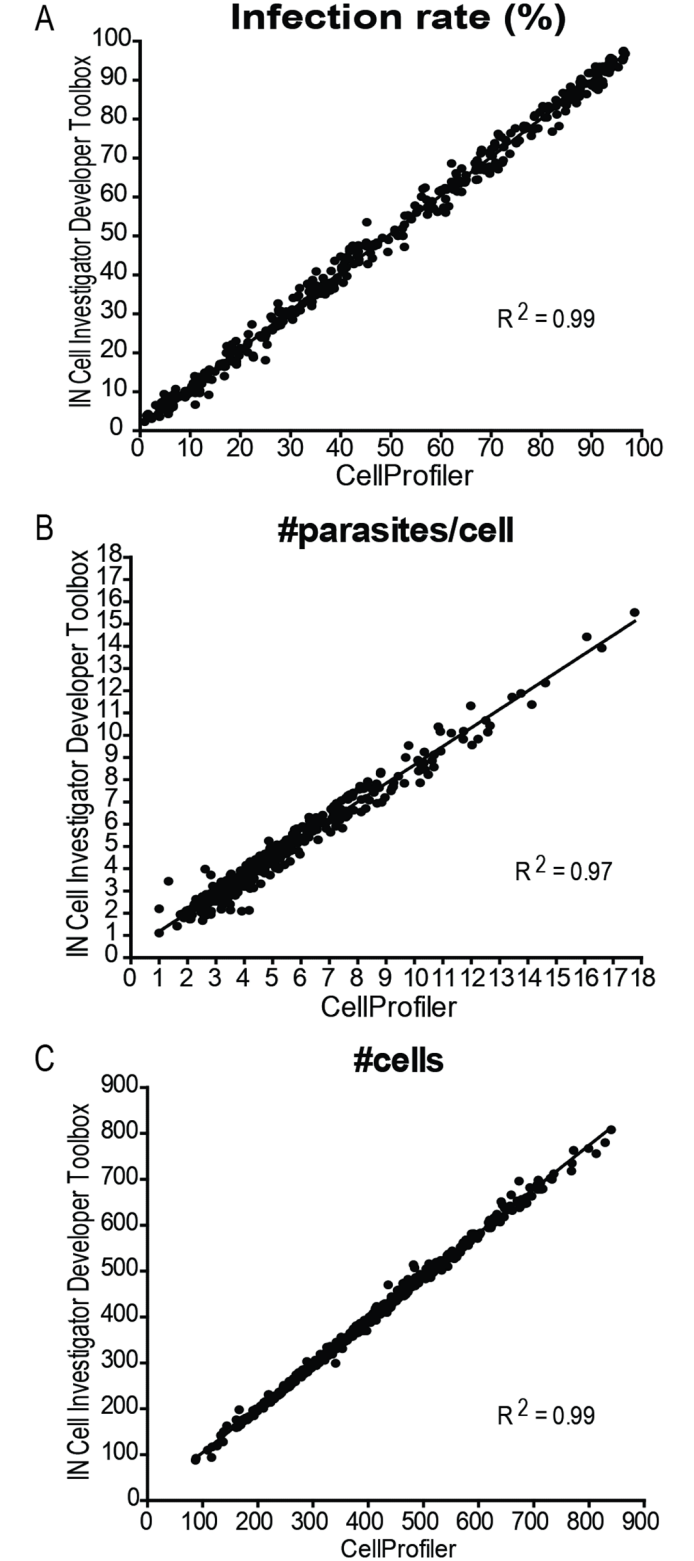
Correlation between IN Cell Investigator Developer Toolbox and CellProfiler image analysis protocols. (A) Infection rate, (B) number of parasites per infected cell, and (C) the total number of cells as compared using both image analysis protocol. Each dot represents the result of a single image.

The CellProfiler image analysis pipeline was additionally compared with the IN Cell Investigator Developer Toolbox protocol. Analysis of 382 images with both workflows indicated a high degree of correlation between them (Fig 7). The infection rate (Fig 7A), the number of parasites per macrophage (Fig 7B), as well as the total number of macrophages (Fig 7C) were similar in both outputs (R^2^>0.97).

### Pharmacological validation of IN Cell Investigator Developer Toolbox and Cell Profiler workflows

**Fig 8:**
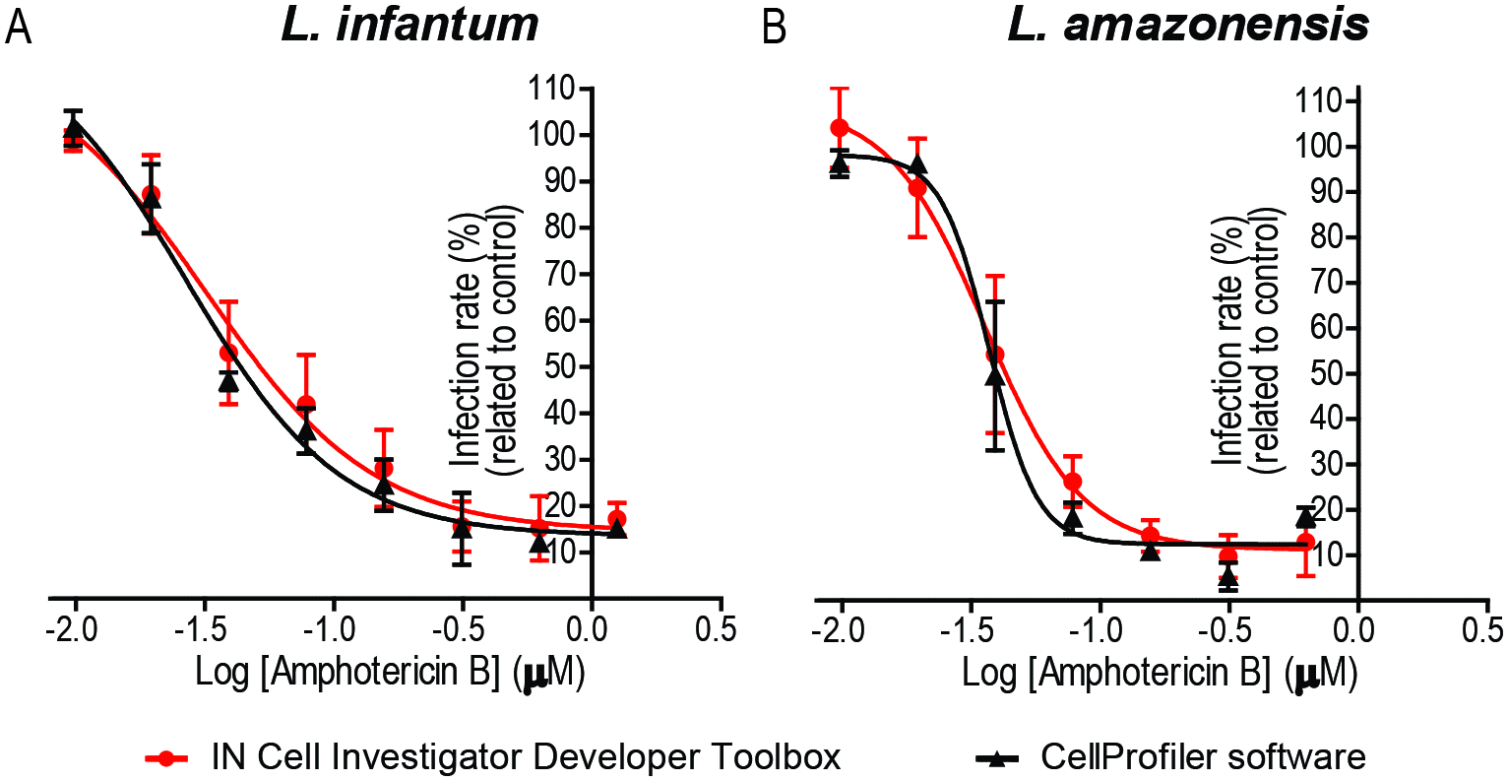
Anti-*Leishmania* activity of amphotericin B assessed with IN Cell Investigator Developer Toolbox and CellProfiler. Dose response curves of (A) *L*. *infantum* or (B) *L*. *amazonensis*. IN Cell Investigator Developer Toolbox (red line) and CellProfiler (black line). Values represent the mean and standard deviation of at least two independent experiments.

Since facilitating anti-*Leishmania* drug testing was a major driving force towards implementation of the IN Cell Investigator Developer Toolbox and CellProfiler protocols, we evaluated their pharmacological relevance. For this, we infected BMDM with *L*. *infantum* and *L*. *amazonensis* and treated the monolayers with amphotericin B, an anti-*Leishmania* reference drug that acts by disrupting parasite membranes and, hence, translates in a rapid decrease in amastigote number [18,19,22,23]. Images acquired at the end of the experiments were then analyzed following both methodologies, extracting both the percentages of infection and the number of amastigotes per infected macrophage. The results, obtained for both *Leishmania* species, are depicted in Fig 8. The IC_50_ values calculated from those data (0.037±0.012 and 0.024±0.010μM for *L*. *infantum* according to IN Cell Investigator Developer Toolbox and CellProfiler, respectively, and 0.037±0.012 and 0.039±0.004μM for *L*. *amazonensis* in the same situations) were in concordance with published results [24].

In short, the image analysis protocols developed using the IN Cell Investigator Developer Toolbox and the CellProfiler software enable high throughput and reliable determination of the number of infected macrophages and of parasites per infected macrophage, independently of the *Leishmania* species used.

Both image analysis workflows, IN Cell Investigator Developer Toolbox protocol (S1 Appendix) and CellProfiler pipeline (S2 Appendix), can be freely downloaded from https://github.com/andrefilipemaia/intramacrophagic_parasites_counter.

## Discussion

Drug discovery for infectious diseases inevitably requires the capacity to triage large numbers of chemical compounds against microbial pathogens, a process simplified by the existence of adequate high throughput phenotypic methodologies. In the case of diseases caused by *Leishmania* parasites, previously developed HTS assays often used promastigotes and axenic amastigotes [22,25–27]. However, in spite of enabling cheap, fast and simple protocols, screening against these parasite forms in many instances lead to false positive and negative hits [18,21,28]. For instance, apparent leishmanicidal compounds will in fact lack clinical relevance if unable to cross host cell membranes or if their target molecule is not expressed by intracellular amastigotes [29–31]. Triage based on axenic parasite forms will exclude chemicals of interest whenever these require host cell metabolism for activity, or when their parasiticidal capacity is promoted not by the compound itself but by its ability to induce an inflammatory state in infected macrophages. For these reasons, recent HTS assays have favored the use of the more physiologically relevant intracellular amastigotes, albeit, with two exceptions [19,32], resorting to macrophage cell lines [18,20,21,23]. In order to mimic as closely as possible an *in vivo* infection, the present assay was established using primary macrophages derived from murine bone marrow. Although optimized here for 96-well plates, with appropriate equipment, the BMDM differentiation protocol can be adapted to 384 or 1536 well plates allowing screening of larger libraries with a relatively low number of animals. The methodology developed in this report is, in addition, a high content analysis (HCA) procedure that quantifies intracellular amastigotes based on optical microscopy. An important feature of microscopy-based methods, when compared to indirect methodologies like luminescence/fluorescence-plate reading approaches, is that they enable visualization and determination of the actual parasite number. Previous studies [21,32] employed genetically modified parasites expressing a reporter gene such as green fluorescence protein (GFP) or DsRed2 molecule to facilitate parasite identification. However, this approach has the immediate drawback that such organisms are no longer wild-type and the reporter genes may interfere with drug screening output by decreasing strain virulence [18,33]. Furthermore, genetic manipulation steps would be required every time a new species or strain is used. The protocol developed in this study overcomes these limitations and can also be used in functional analysis studies of *Leishmania* requiring transgenic or knockout mutants.

Determination of the number of macrophages and intracellular amastigotes was first automated with the IN Cell Investigator Developer Toolbox, a proprietary software accompanying our high content microscope, IN Cell Analyzer 2000. However, many laboratories performing *Leishmania* drug development studies do not have easy access to HCS-microscopes and this specific software. In such cases, drug screening ability is limited by the time required for image acquisition and analysis. In order to simplify the latter, we proposed the CellProfiler image analysis pipeline whose correlation with IN Cell Investigator Developer protocol was very high (R^2^>0.97). Since the activity of some drugs (*eg* miltefosine) can differ between *Leishmania* spp., candidate compounds ought to be screened *in vitro* against several species [34,35]. Both software workflows employed in this study are suitable for addressing this issue as they were optimized and validated with *L*. *infantum* and *L*. *amazonensis*. The former parasite is representative of species maturing in small individual parasitophorous vacuoles, while *L*. *amazonensis* represents *Leishmania* spp. inhabiting communal vacuoles housing several amastigotes, a factor that dramatically changes host cell morphology. The use of *L*. *infantum* axenic amastigotes to infect BMDM relates to the high infection rates that are possible to obtain with this parasite form. Unfortunately, we are not able to cultivate *L*. *amazonensis* axenic amastigotes and, as such, resorted to stationary phase promastigotes. Both image analysis workflows are comparable in terms of performance with manual counting; however, in some circumstances, adjustment of specific image analysis settings might be required for maximum performance. This is the case, for instance, when analyzing compounds affecting host cell nucleus morphology.

In conclusion, this work presents a new methodology for identification of novel therapeutic drugs for leishmaniasis in two image analysis platforms, IN Cell Investigator Developer Toolbox and the free open source Cell Profiler. This workflow should enable fast and standardized analysis of microscopy images from *Leishmania*-infected macrophages in any laboratory.

## Supporting information

S1 Fig Detection of intramacrophagic *L*. *amazonensis* parasites using IN Cell Investigator Developer Toolbox and CellProfiler. Raw image from (A) DAPI and (B) HCS CellMask^™^ Deep Red imaging channels acquired with IN Cell Analyzer 2000 microscope. (C) Final processed image obtained from IN Cell Investigator Developer Toolbox, showing macrophage nuclei (blue line), cell boundaries (yellow line) and parasites (green line). (D) CellProfiler final processed image showing BMDM nuclei expansion (light green line), cell boundary (pink line) and parasites (dark green line). Scale bar, 20μm.
(TIF)

S1 Appendix IN Cell Investigator Developer Toolbox protocol. (XEAP)

S2 Appendix CellProfiler pipeline. (CPPIPE)
