# Supplementary figures and images for: Development of an automated image analysis protocol for quantification of intracellular forms of Leishmania spp
Source: PLoS One. 2018 Aug 2;13(8):e0201747. doi: 10.1371/journal.pone.0201747 (PMC6072083; doi:10.1371/journal.pone.0201747)

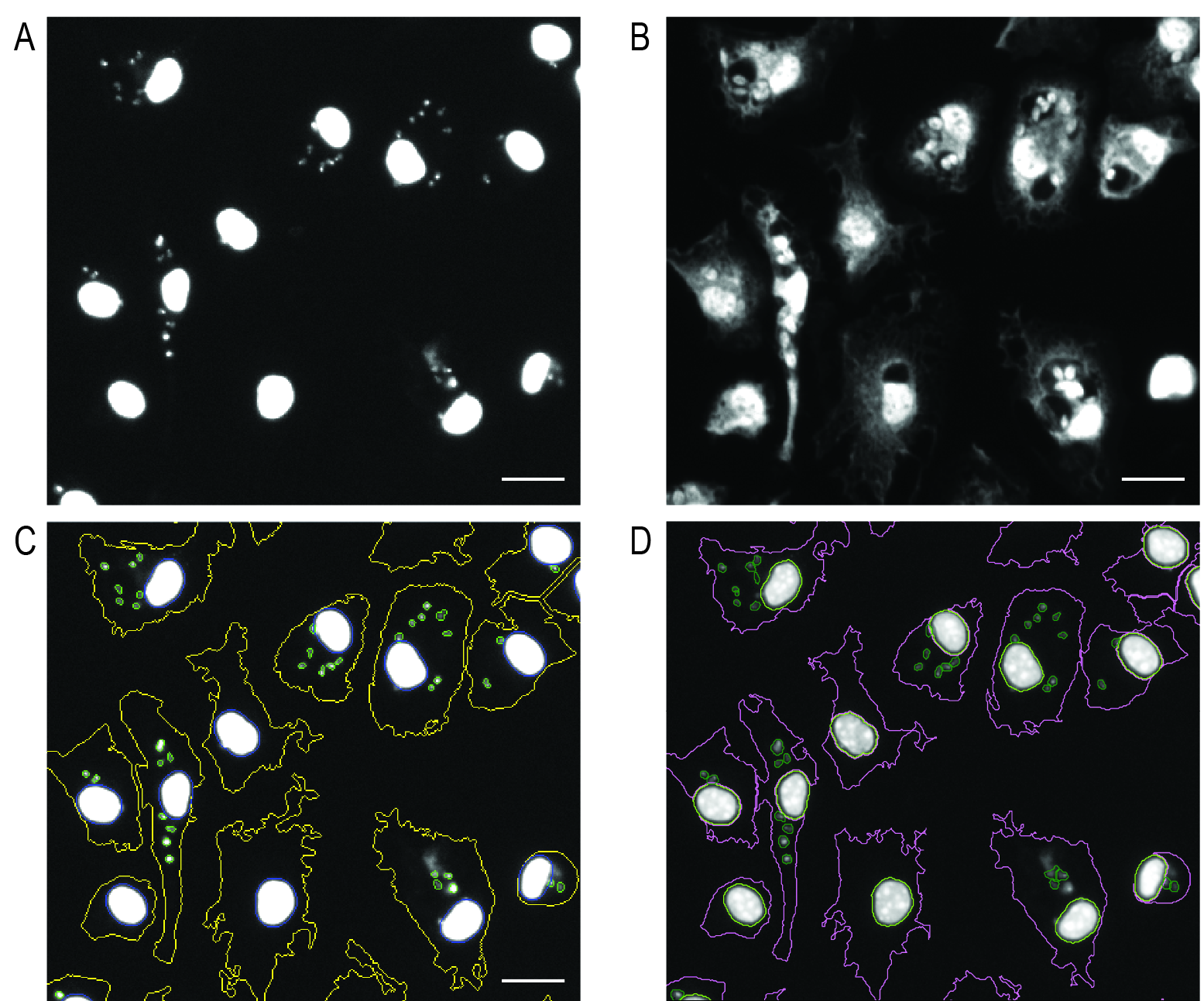

Supplement: S1 Fig — Raw image from (A) DAPI and (B) HCS CellMask™ Deep Red imaging channels acquired with IN Cell Analyzer 2000 microscope. (C) Final processed image obtained from IN Cell Investigator Developer Toolbox, showing macrophage nuclei (blue line), cell boundaries (yellow line) and parasites (green line). (D) CellProfiler final processed image showing BMDM nuclei expansion (light green line), cell boundary (pink line) and parasites (dark green line). Scale bar, 20μm. (TIF) [file pone.0201747.s001.tif]
